# Photovoltage memory effect in a portable Faradaic junction solar rechargeable device

**DOI:** 10.1038/s41467-022-30346-z

**Published:** 2022-05-10

**Authors:** Pin Wang, Mengfan Xue, Dongjian Jiang, Yanliang Yang, Junzhe Zhang, Hongzheng Dong, Gengzhi Sun, Yingfang Yao, Wenjun Luo, Zhigang Zou

**Affiliations:** 1 Eco-materials and Renewable Energy Research Center (ERERC), Jiangsu Key Laboratory for Nano Technology, National Laboratory of Solid State Microstructures and Department of Physics, Nanjing University, Nanjing, 210093 China; 2 College of Engineering and Applied Sciences, Nanjing University, Nanjing, 210093 China; 3 Key Laboratory of Flexible Electronics (KLOFE) & Institute of Advanced Materials (IAM), Nanjing Tech University, Nanjing, 211816 China

**Keywords:** Photochemistry, Solar energy

## Abstract

Two-electrode solar rechargeable device is one of the promising technologies to address the problem of solar energy storage in large scale. However, the mechanism of dark output voltage remains unclear and the low volumetric energy density also limits its practical applications. Herein, we report that a Si/CoO_x_/KBi_(aq)_/MnO_x_ Faradaic junction device exhibits a photovoltage memory effect, that is, the dark output voltage can precisely record the value of the photovoltage in the device. To investigate the mechanism of the effect, we develop an open circuit potential method to real-time monitor the photo charge and dark discharge processes in the Faradaic junction device. This effect leads to minimized interface energy loss in the Faradaic junction device, which achieves much higher performances than the devices without the effect. Moreover, we realize a portable device with a record value of the dark volumetric energy density (∼1.89 mJ cm^−3^) among all reported two-electrode solar rechargeable devices. These results offer guidance to improve the performance of a solar rechargeable device and design other photoelectric devices for new applications.

## Abstract (web summary)

A Faradaic junction solar rechargeable device is one of the promising technologies to address the problem of solar energy storage but the working mechanism remains unclear. Here, the authors report a photovoltage memory effect in a portable Si/CoO_x_/KBi_(aq)_/MnO_x_ Faradaic junction device.

## Introduction

Solar energy provides an environmentally benign alternative to fulfill the increasing global energy demand^1^. During the past decades, photovoltaic devices can convert solar energy into electricity and have attracted enormous attention^2–4^. However, the generated electricity needs to be stored to balance the intermittence of solar irradiance. In previous studies, solar supercapacitors or solar batteries can address this problem by simultaneous conversion and storage of solar energy^5–13^, which are typically with three-electrode or four-electrode configuration by the integration of solar cells and energy storage units. Nevertheless, these devices suffer from complicated structures and high cost, which limit their applications in a large scale^14,15^. Recently, some facile two-electrode devices based on Faradaic junction photoelectrodes, such as Fe_2_O_3_/Ni(OH)_2_ and Si/WO_3_, have been also reported (Supplementary Fig. 1a)^16–21^. A Faradaic junction is a semiconductor/Faradaic material junction with coupled electron-ion transfer at the interface^18,19^, which can directly store photo-generated carriers into the Faradaic material when the semiconductor is illuminated^21^. According to previous study, the photovoltage in a Faradaic junction device is equal to the difference between the Fermi level of a semiconductor and the onset potential of a Faradaic material^18,22^. However, it is still unclear about the influence factors on a dark output voltage, which is detrimental for designing a high-performance Faradaic junction device. In addition, these devices also show undesirable bulky volume, further hindering their practical applications. Therefore, it is significant to clarify the generation mechanism of the dark output voltage and construct a two-electrode solar rechargeable device with high volumetric energy density.

Herein, by assembling an n-Si/CoO_x_ Faradaic junction photoelectrode and a MnO_x_ counter electrode into a two-electrode device (Supplementary Fig. 1b), we find a photovoltage memory effect with the dark output voltage of the Faradaic junction device equal to the photovoltage. Further studies suggest that a Faradaic junction device with the photovoltage memory effect can minimize the interface energy loss and leads to much higher output performance of the device than one without the effect. In order to investigate the mechanism on the photovoltage memory effect, we develop an open circuit potential method to real-time monitor the photo charge and dark discharge processes of a photoelectrode and a counter electrode, respectively. According to the method, we obtain a prerequisite for photovoltage memory effect in a Faradaic junction solar rechargeable device. Moreover, by using a semitransparent counter electrode instead of an opaque one, we remarkably simplify the configuration of the device and reduce the volume, which achieves the highest dark volumetric energy density among all reported two-electrode solar rechargeable devices.

## Results

### Coupled electron-ion transfer in a Si/CoO_x_ photoelectrode and a MnO_x_ counter electrode

**Fig. 1:**
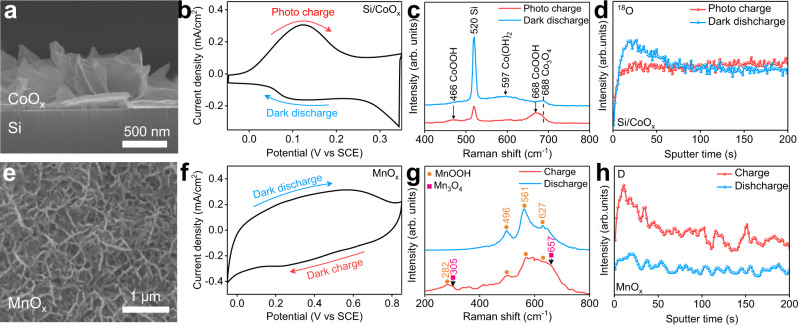
Characterization of Si/CoO_x_ photoelectrodes and MnO_x_ counter electrodes. **a** Cross-sectional SEM of Si/CoO_x_. **b** CV curve of a Si/CoO_x_ photoelectrode at the scan rate of 10 mV s^−1^ during photo charge and dark discharge. **c** Raman spectra of Si/CoO_x_. **d** TOF-SIMS spectra of ^18^O depth profile in Si/CoO_x_. **e** Surface SEM image of MnO_x_. **f** CV curve of a MnO_x_ counter electrode at the scan rate of 10 mV s^−1^ in the dark. **g** Raman spectra of MnO_x_. **h** TOF-SIMS spectra of D depth profile in MnO_x_. Light source: 1 Sun of simulated solar illumination by a Xe lamp with AM 1.5 G filter (100 mW cm^−2^), electrolyte: KBi aqueous solution (0.2 M KOH and 0.4 M H_3_BO_3_) with pH = 9.

We obtained a photoelectrode by electrodepositing a CoO_x_ layer on monocrystalline n-type silicon and then calcinating in air at 150 ^o^C for 1 h. Scanning electron microscopy (SEM) images show that CoO_x_ nanosheets vertically grow on the surface of the Si and the thickness of the CoO_x_ film is 600 ~ 700 nm (Fig. 1a and Supplementary Fig. 2). We then performed the cyclic voltammogram (CV) curve of Si/CoO_x_ photoelectrode in a three-electrode setup in the dark and under illumination (Supplementary Fig. 3 and Fig. 1b). The Si/CoO_x_ indicates negligible current in the dark, whereas obvious photoanodic current is observed at the potential range of −0.05 V_SCE_ to 0.35 V_SCE_ under illumination. When the light is off, symmetrical dark cathodic current is obtained. These results suggest that Si/CoO_x_ can be photo-charged and dark discharged at the same potential range. Moreover, a galvanostatic charge-discharge (GCD) curve exhibits continuously adjustable potentials are obtained in the Si/CoO_x_^19,23^ (Supplementary Fig. 4), which is a prerequisite for a bias-free two-electrode solar rechargeable device^19^. To investigate the electrode reaction process of a Si/CoO_x_ Faradaic junction, Raman spectroscopy was carried out (Fig. 1c)^24^. For the dark discharged sample, Raman peaks at 520, 597 and 688 cm^−1^ are observed, which correspond to Si, Co(OH)_2_ and Co_3_O_4_, respectively^25,26^. When the Si/CoO_x_ is photo charged, a Raman peak at 597 cm^−1^ of Co(OH)_2_ disappears and two new peaks appear at 466 and 668 cm^−1^, which are assigned as CoOOH^26^. UV-Vis spectra also confirm that the reflectance of Si/CoO_x_ decreases after Co(OH)_2_ is oxidized into CoOOH (Supplementary Fig. 5). X-ray photoelectron spectroscopy (XPS) was used to further investigate the Faradaic reaction in Si/CoO_x_ (Supplementary Fig. 6). After photo charge, the intensities of satellite peak of Co^2+^ and OH^−^ species decrease remarkably, and Co 2*p* core level emission shifts to higher binding energy^27^, which suggest that Co is oxidized and electron transfer happens during photo charge. In order to further study ion transfer in the Si/CoO_x_ heterojunction, we performed isotope-labeling experiments by using H_2_^18^O as the solvent and time-of-flight secondary-ion mass spectrometry (TOF-SIMS) to analyze the depth profiles of ^18^O ions (Fig. 1d). Compared with the photo-charged Si/CoO_x_, the intensity of ^18^O ions in the sample after dark discharge increases obviously at the depth with sputtering time < 100 s, which suggests O^2−^ ions exchange between CoO_x_ and H_2_^18^O. Therefore, coupled electron and ion transfer happens in Si/CoO_x_, following a possible Faradaic reaction, Co(OH)_2_ + h^+^ +OH^−^ ↔ CoOOH + H_2_O.

The counter electrode was prepared by electrodepositing MnO_x_ on fluorine-doped tin oxide (FTO) substrate. SEM image indicates that the MnO_x_ film is flake-like microstructure (Fig. 1e). The MnO_x_ electrode exhibits nearly symmetric shape at a potential window of −0.05 ~ 0.85 V_SCE_ (Fig. 1f) and linearly adjustable potential in GCD curve (Supplementary Fig. 7), which indicates typical capacitive features^23^. To further study the reaction process on MnO_x_ electrode, we performed Raman spectra of the electrode during charge and discharge process in the dark (Fig. 1g), similar to the Si/CoO_x_ photoelectrode. Three Raman peaks at 496, 561 and 627 cm^−1^ in the discharged MnO_x_ are assigned to MnOOH^28^. After dark charge, the peak intensities decrease and two new Raman peaks of Mn_3_O_4_ are observed at 305 and 657 cm^−1^ ^29^. Moreover, a new Raman peak at 282 cm^−1^ of MnOOH also appears after dark charge^28^, which possibly comes from the disproportionation reaction of Mn_3_O_4_^30^. Transmission electron microscopy (TEM) images also show that the lattice spacings of MnOOH are observed in the discharged sample, and the lattice spacings of Mn_3_O_4_ are observed after dark charge (Supplementary Fig. 8). To investigate the reaction process of ions in the MnO_x_ electrode, we used D_2_O as the solvent and TOF-SIMS to analyze the depth profiles of D ions (Fig. 1h). The intensity of D ions increases remarkably after charge and decreases to negligible after discharge, which suggests that Faradaic reactions intercalation and de-intercalation of D ions (3MnOOH + e^−^ + H^+^ ↔ Mn_3_O_4_·2H_2_O)^31^ happen in MnO_x_ electrode.

### Photovoltage memory effect and performance of Faradaic junction solar rechargeable devices

**Fig. 2:**
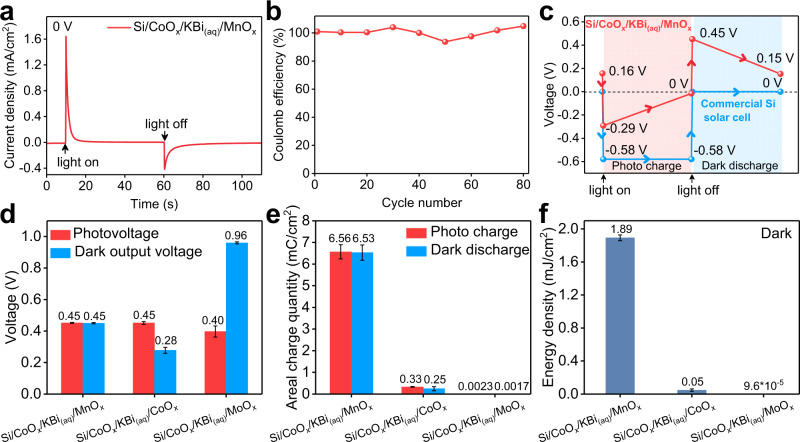
Photoelectrochemical properties of Faradaic junction devices. **a** *I*–*t* curve during photo charge and dark discharge under zero bias of the Si/CoO_x_/KBi_(aq)_/MnO_x_ device. **b** Coulomb efficiency of the Si/CoO_x_/KBi_(aq)_/MnO_x_ device during photo charge and dark discharge cycles. **c** Open circuit voltages of the Si/CoO_x_/KBi_(aq)_/MnO_x_ Faradaic junction device and a commercial Si solar cell, and the photo charge and dark discharge were carried out in the Faradaic junction device by i-t curves without bias. **d** Photovoltages and dark output voltages of Si/CoO_x_/KBi_(aq)_/MnO_x_, Si/CoO_x_/KBi_(aq)_/CoO_x_ and Si/CoO_x_/KBi_(aq)_/MoO_x_ devices. **e** Areal charge quantities of photo charge and dark discharge in the three devices. **f** Dark output energy density of the three devices. The results in Fig. 2d–f show average values with the standard deviation as the error bar. Light source: 1 Sun of simulated solar illumination by a Xe lamp with AM 1.5 G filter (100 mW cm^−2^), electrolyte: KBi aqueous solution (0.2 M KOH and 0.4 M H_3_BO_3_) with pH = 9.

We then fabricated a two-electrode solar rechargeable device by a Si/CoO_x_ photoelectrode and a MnO_x_ counter electrode (Supplementary Fig. 1b). The I-t curve of Si/CoO_x_/KBi_(aq)_/MnO_x_ under photo charge and dark discharge without bias was measured and the result is shown in Fig. 2a. An initial photocurrent density of 1.64 mA cm^−2^ is observed and then the photocurrent decays rapidly in the device under illumination, which is similar to the charging behavior of a conventional supercapacitor under voltage-constant mode^32^. The anodic photocurrent suggests electrons flow from the Si/CoO_x_ photoelectrode to the MnO_x_ counter electrode through the external circuit. The photo-generated holes and electrons are stored in CoO_x_ and MnO_x_, respectively. When the light is off, the device indicates an initial opposite current density of −0.40 mA cm^−2^ without bias, which suggests that the stored charges can go back. The asymmetric current behavior between photo charge and dark discharge comes from higher electronic conductivity^33^ and lower charge transfer resistance after illumination (Supplementary Fig. 9 and Supplementary Table 1). Moreover, the cycle stability of the device during photo charge and dark discharge was also measured and the results are shown in Fig. 2b and Supplementary Fig. 10. The initial coulomb efficiency is about 100% and remains stable after 80 cycles, which suggests that side reactions are negligible during photo charge and dark discharge. Therefore, the Si/CoO_x_/KBi_(aq)_/MnO_x_ realizes reversible photo charge and dark discharge under zero bias.

We further investigated the relationship between the dark output voltage and the photovoltage of the Faradaic junction device (Fig. 2c). The Si/CoO_x_/KBi_(aq)_/MnO_x_ device indicates a residual open circuit voltage (V_R_) of 0.16 V in the dark, which comes from different dark equilibrium potentials (V_E_) of the photoelectrode and the counter electrode. When light is on, photovoltage is generated in the Si/CoO_x_ Faradaic junction, leading to a reverse open circuit voltage of −0.29 V. Therefore, the photovoltage can be calculated as 0.45 V. During photo charge process in short circuit, the photo-generated holes are stored in the CoO_x_ layer and photo-generated electrons in the MnO_x_ counter electrode, respectively, which results in an open circuit voltage decreasing gradually^19^. When the potential of the photoelectrode is equal to that of the counter electrode, the photo charge process ends and the open circuit voltage is 0 V. And then the light is off, the Si/CoO_x_/KBi_(aq)_/MnO_x_ device exhibits a dark output voltage of 0.45 V, which is the same value as the photovoltage in the Si/CoO_x_ Faradaic junction mentioned above. In contrast, a commercial silicon solar cell indicates a photovoltage of 0.58 V, but a negligible dark output voltage. Therefore, the Faradaic junction device shows a photovoltage memory effect, which is intrinsically different from the photovoltaic effect in a classic p-n junction^34^. Furthermore, neither such photovoltage memory effect is observed in previously reported three-electrode or four-electrode solar rechargeable devices, in which self-discharging voltages are usually lower than photovoltages^35–39^. It is possibly due to the mismatch of operation voltages between solar cells and energy storage units^35,36^, or high internal resistance^37–39^.

In order to investigate the effects of the photovoltage memory effect on the performance, we also replaced the MnO_x_ counter electrode with CoO_x_ or MoO_x_ as reference. After optimizing the loading amount of the Faradaic materials (Supplementary Fig. 11), the photovoltages and dark output voltages of the devices with MnO_x_, CoO_x_ and MoO_x_ counter electrodes are measured and shown in Supplementary Fig. 12a and Fig. 2d, respectively. Different from Si/CoO_x_/KBi_(aq)_/MnO_x_, the device with CoO_x_ counter electrode indicates lower dark output voltage than the photovoltage, while Si/CoO_x_/KBi_(aq)_/MoO_x_ device shows a higher dark output voltage than the photovoltage (The reasons are clarified in Supplementary Figs. 18–21). Therefore, the devices with CoO_x_ and MoO_x_ counter electrodes do not have the photovoltage memory effect. The charge quantities of photo charge and dark discharge in the three devices are calculated by integrating I-t curves^23^ in Supplementary Fig. 12b. The Si/CoO_x_/KBi_(aq)_/MnO_x_ shows much higher stored charge quantity than the Si/CoO_x_/KBi_(aq)_/CoO_x_ and Si/CoO_x_/KBi_(aq)_/MoO_x_ (Fig. 2e), which leads to the Si/CoO_x_/KBi_(aq)_/MnO_x_ indicating the energy density over 30 times and 1.9*10^4^ times higher than the devices with CoO_x_ and MoO_x_ counter electrodes, respectively (Fig. 2f).

### A mechanism of the photovoltage memory effect

**Fig. 3:**
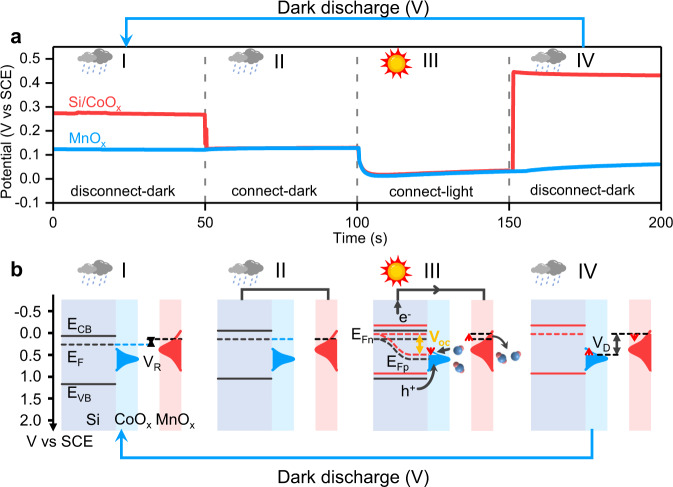
A mechanism of the photovoltage memory effect in a Si/CoO_x_/KBi_(aq)_/MnO_x_ Faradaic junction device. **a** OCPs of a Si/CoO_x_ photoelectrode and a MnO_x_ counter electrode under illumination and in the dark. The OCPs were measured under disconnected and connected modes. Light source: 1 Sun of simulated solar illumination by a Xe lamp with AM 1.5 G filter (100 mW cm^−2^), electrolyte: KBi aqueous solution (0.2 M KOH and 0.4 M H_3_BO_3_) with pH = 9. **b** Energy band diagrams of Si/CoO_x_/KBi_(aq)_/MnO_x_ at different working stages. E_VB_ and E_CB_ are the valence band and conduction band of a semiconductor, respectively; E_Fn_ and E_Fp_ represent the quasi-Fermi levels of electrons and holes, respectively; *V*_R_ is a residue voltage between a photoelectrode and a counter electrode; *V*_oc_ represents a photovoltage and V_D_ represents a dark output voltage.

Since a Faradaic junction device with the photovoltage memory effect indicates higher performance than the devices without the effect, it is very significant to study the mechanism of the photovoltage memory effect in the Si/CoO_x_/KBi_(aq)_/MnO_x_ device. We therefore developed an open circuit potential (OCP) method^40^ to real-time monitor the potentials of the photoelectrode and the counter electrode at different working stage (Fig. 3a). The measurement details are shown in Supplementary Fig. 13. When an electrode is immersed into the electrolyte, electrochemical equilibrium is established between the electrode surface and the environment. The OCP method can measure the equilibrium potential with respect to a reference electrode. The voltage of a device is the difference between the equilibrium potentials of the two electrodes^41^. From Fig. 3a, the Si/CoO_x_ photoelectrode and the MnO_x_ counter electrode are disconnected in the dark (Stage I), the OCPs of Si/CoO_x_ and MnO_x_ are about 0.27 V_SCE_ and 0.13 V_SCE_, respectively. When Si/CoO_x_ and MnO_x_ are connected directly by a Cu wire in the dark (Stage II), the OCP of MnO_x_ does not change, while the OCP of Si/CoO_x_ is adjusted to the same potential of MnO_x_ due to much higher density of storable charge in MnO_x_ than Si/CoO_x_ in the dark (Supplementary Fig. 14). When the light is on (Stage III), the OCPs of Si/CoO_x_ and MnO_x_ both decrease gradually from 0.13 V_SCE_ to 0.02 V_SCE_, which suggests that the photo charge process happens between the two electrodes. According to Fig. 1b, f, the Si/CoO_x_ photoelectrode and the MnO_x_ counter electrode cannot drive water splitting reactions in the potential range of 0.13 V_SCE_ to 0.02 V_SCE_, which further confirms that side reactions are negligible under zero bias. The results can understand the nearly 100% coulomb efficiency in Fig. 2b. When the light is off and the two electrodes are disconnected from each other (Stage IV), the OCP of Si/CoO_x_ jumps to a positive potential of 0.45 V_SCE_, while the OCP of MnO_x_ remains the same potential at Stage III, which leads to the output dark voltage of 0.43 V. When the photo-charged device is discharged in the dark (Stage V in Fig. 3a), the potentials of the photoelectrode and the counter electrode can recover to the initial values.

We then plotted the band diagrams of the Faradaic junction device under disconnected and connected modes (Fig. 3b). The energy band positions of the Si semiconductor and the Faradaic potential windows of CoO_x_ and MnO_x_ were measured by electrochemical methods. A bare Si indicates a flat-band potential of −0.11 V_SCE_ after anodic cycling activation process due to the formation of SiO_x_ on the surface^42^ (Supplementary Fig. 15). The Faradaic potential windows of MnO_x_ and CoO_x_ on FTO were measured by CV and GCD methods, as shown in Fig. 1f, Supplementary Figs. 7 and 16. In the Si/CoO_x_ Faradaic junction, the Fermi level of Si is adjusted by CoO_x_ through interface charge transfer and an equilibrium potential is established (Fig. 3b, Stage I). As mentioned above, the Si/CoO_x_/KBi_(aq)_/MnO_x_ device indicates a residue voltage (V_R_) of 0.16 V (Fig. 2c), which comes from the different OCPs of Si/CoO_x_ and MnO_x_. When Si/CoO_x_ and MnO_x_ are connected by a Cu wire in the dark, the Fermi level of Si is adjusted to the equilibrium potential of MnO_x_ (Fig. 3b, Stage II). After the light is on (Fig. 3b, Stage III), photo-generated holes in Si oxidize Co^2+^ into Co^3+^, which shifts the potential of CoO_x_ positively. A photovoltage (V_oc_) is generated in a Si/CoO_x_ heterojunction, which is intrinsically different from the photovoltage from a buried p-n junction in our previous study^19^. When the hole quasi-Fermi level in Si is the same with the potential of CoO_x_, the photo charge process ends. On the other hand, photo-generated electrons transfer from Si to the MnO_x_ counter electrode by a Cu wire. Since the electron quasi-Fermi level in Si always keeps the same potential with MnO_x_ due to the short circuit connection, the photo-generated electrons shift the potential of MnO_x_ negatively, as well as the Fermi level of Si. We experimentally observe the quasi-Fermi levels of both electron and hole in a semiconductor at the same time. The results are in good agreement with our previous study^43^, in which a Faradaic junction indicates a characteristic of isoenergetic interfacial charge transfer and can minimize the interface energy loss. Therefore, the electron and hole quasi-Fermi levels in Si under illumination are exactly recorded by MnO_x_ and CoO_x_, respectively. Both MnO_x_ and CoO_x_ indicate high Faradaic capacitance, the potentials of MnO_x_ and CoO_x_ after photo charge can be stored even when the light is off (Fig. 3b, Stage IV), which is the reason for the photovoltage memory effect in the Faradaic junction device. Based on above analysis, we plot a working mechanism schematic of the device with the photovoltage memory effect (see Supplementary Fig. 17). Moreover, by comparing with the devices without the photovoltage memory effect (Supplementary Figs. 18–21), we conclude that the high performance of Si/CoO_x_/KBi_(aq)_/MnO_x_ comes from the longer storage time and higher charge quantity of the Faradaic layer than the electric double layer^23,44^.

We further propose a prerequisite to realize the photovoltage memory effect, as shown in Supplementary Fig. 22. When a photoelectrode is connected with a counter electrode in short-circuit in the dark, its Fermi level will be adjusted to the equilibrium potential of the counter electrode since the photoelectrode indicates much lower storable charge density than the counter electrode in the dark. The hole quasi-Fermi level in a semiconductor should locate within the Faradaic potential window of a Faradaic material on the semiconductor for hole storage. On the other hand, the equilibrium potential of a counter electrode should locate within its Faradaic potential window for electron storage. Therefore, to achieve the photovoltage memory effect in a Faradaic junction device, the electron and hole quasi-Fermi levels in a semiconductor should locate within the potential windows of Faradaic materials in the two electrodes, respectively.

### Demonstration of a portable Faradaic junction solar rechargeable device

**Fig. 4:**
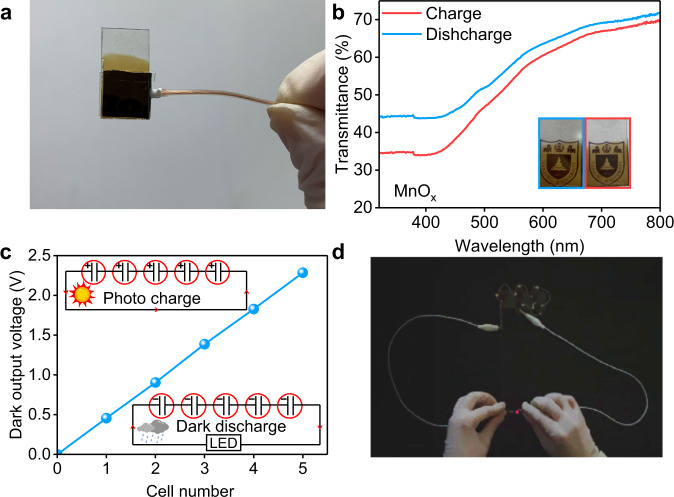
Demonstrations of Faradaic junction solar rechargeable devices. **a** A picture of a portable Si/CoO_x_/KBi_(aq)_/MnO_x_ device with an effective area of about 0.8 cm^2^. **b** UV-Vis spectra of a MnO_x_ counter electrode after charge and discharge in the dark. **c** Dark output voltages of five Si/CoO_x_/KBi_(aq)_/MnO_x_ devices connected in series after photo charge. Inset picture: electrical connection modes of five devices under photo charge and dark discharge. **d** Photograph of five Si/CoO_x_/KBi_(aq)_/MnO_x_ devices connected in series to power a LED after photo charge.

Finally, we developed a portable Si/CoO_x_/KBi_(aq)_/MnO_x_ device with a significantly reduced volume (Fig. 4a and Supplementary Fig. 23). Because the MnO_x_ counter electrode remains semitransparent during charge and discharge (Fig. 4b), it allows sunlight to incident across the counter electrode onto the photoelectrode (Supplementary Fig. 1b). A surlyn membrane was used as separator in the portable device and the electrolyte was injected between the two electrodes. We investigated the effects of the membrane thickness on the performance of the Si/CoO_x_/KBi_(aq)_/MnO_x_ Faradaic junction device and the results are shown in Supplementary Fig. 24. As the thickness increases, the initial charge/discharge current density of the device decreases, which possibly comes from faster mass-transfer in the thinner device. The portable device exhibits a dark volumetric energy density over 30 times higher than the reported device in our previous study^19^ and reaches a record value among all reported two-electrode solar rechargeable devices (Supplementary Table 2). In addition, the Faradaic junction device with the photovoltage memory effect can be used for direct solar energy storage. After illumination, five Si/CoO_x_/KBi_(aq)_/MnO_x_ units connected in series indicates a dark output voltage of 2.3 V (Fig. 4c), which can power a light emitting diode (LED) bulb in the dark under zero bias (Fig. 4d and Supplementary Movie 1). For practical application, the Faradaic junction solar rechargeable device should be further upscaled. For this purpose, substrate ohmic loss, ionic transport resistance, parasitic adsorption of Faradaic materials should be decreased. Some possible methods, such as conductive silver frame as collector^11^, further reducing the distance between the two electrodes and introducing back-side illuminated photoelectrodes^45^, can be used. Moreover, it is also significant to develop large-scale deposition methods for Faradaic materials on the semiconductors and FTO substates^11^.

## Discussion

In summary, we find the photovoltage memory effect in the Si/CoO_x_/KBi_(aq)_/MnO_x_ solar rechargeable device, which achieves the highest dark volumetric energy density among all reported two-electrode solar rechargeable devices. The photovoltage memory effect is only observed in the Faradaic junction device, but not in p-n junction solar cells or three-electrode/four-electrode solar rechargeable devices. The effect in the device comes from the electron and hole quasi-Fermi levels in the Si semiconductor under illumination being exactly recorded by MnO_x_ and CoO_x_, respectively. Moreover, we propose a prerequisite to realize the photovoltage memory effect in a solar rechargeable device, the electron and hole quasi-Fermi levels of a semiconductor within the potential windows of Faradaic materials in the two electrodes, respectively. The two-electrode Faradaic junction device with the photovoltage memory effect can minimize the interface energy loss, which has a potential to achieve higher performance than three-electrode and four-electrode solar rechargeable devices. These results pave an avenue to device integration and provide the possibility to construct other new photoelectric devices.

## Methods

### Preparation of n-Si/CoO_x_ photoelectrodes

Before depositing CoO_x_, phosphorous-doped (100) n-type Si wafers with a resistivity of 1–10 Ω cm^−1^ (Shunsheng electronics, China) were cut into 1 × 1 cm^2^. The wafers were ultrasonically cleaned in acetone, ethanol, and deionized water in turn for 10 min, and then were immersed into mixture solution of H_2_SO_4_ and H_2_O_2_ (3:1, v/v) for 10 min, and cleaned by deionized water. Indium particles were welded onto the n-type Si to form ohmic back contact and a Cu wire was attached to the indium for (photo-)electrochemical experiments. In order to prevent leakage current, the lateral and back sides of n-type Si were sealed by silica glue. The sealed Si photoelectrode was immersed in 5% HF solution for 30 s to remove the native surface SiO_2_ layer. An chronopotentiometry technique was used to electrodeposit CoO_x_ on the surface of Si in 0.1 M Co(NO_3_)_2_ aqueous solution with - 0.1 mA cm^−2^. Unless otherwise specified, the loading amount of CoO_x_ on Si was 30 mC cm^−2^. In the electrodeposition, a Si photoelectrode, a saturated calomel electrode (SCE) and a graphite rod were used as a working electrode, a reference electrode, and a counter electrode, respectively.

### Preparation of MnO_x_, CoO_x_ and MoO_x_ counter electrodes

An electrodeposition technique was used to coat MnO_x_, CoO_x_, MoO_x_ on fluorine-doped tin oxide (FTO) substrates in a three-electrode cell. A FTO substrate, a saturated calomel electrode (SCE) and a graphite rod were used as a working electrode, a reference electrode, and a counter electrode, respectively. Aqueous solution of 0.1 M (CH_3_COO)_2_Mn, 0.1 M Co(NO_3_)_2_ and 0.05 M (NH_4_)_6_Mo_7_O_24_·4H_2_O were used as the electrolytes for the deposition of MnO_x_, CoO_x_ and MoO_x_, respectively. MnO_x_ and CoO_x_ were electrodeposited by chronopotentiometry technique at +0.5 mA cm^−2^ for 200 s and −0.1 mA cm^−2^ for 300 s on FTO substrates, respectively. MoO_x_ was electrodeposited on an FTO substrate at a constant potential of −0.8 V_SCE_ with deposition charge of 100 mC cm^−2^.

### Assembly of solar rechargeable devices

The Si/CoO_x_ was used as a photoelectrode. MnO_x_, CoO_x_ and MoO_x_ were used as counter electrodes. The electrolyte was KBi solution (0.2 M KOH and 0.4 M H_3_BO_3_) with pH = 9. Before assembling the device, Si/CoO_x_ was activated by scanning from −0.3 V_SCE_ to 0.6 V_SCE_ for 25 cycles under illumination. A surlyn membrane with 60, 90 and 160 μm thicknesses was used as a separator between the Si/CoO_x_ photoelectrode and the counter electrode, and then was dried in an Oven for 10 min to fix the two electrodes. Finally, the electrolyte was injected into the gap between the two electrodes.

### Characterization of samples

The morphologies of the samples were characterized by scanning electron microscopy (SEM, Nano Nova S230) with an accelerating voltage of 15 kV. The Raman spectroscopy (Horiba T64000, excitation wavelength ~ 532 nm) and transmission electron microscopy (TEM, Tecnai G2 F20) were used to characterize the samples during charge and discharge process. Transmittance and reflectance of the films were analyzed by using ultraviolet-visible-near infrared spectroscopy (UV-Vis-NIR, Perkin Elmer Lambda 950). X-ray photoelectron spectroscopy (XPS) was performed on a K-Alpha instrument operating with an Al Kα X-ray source. The binding energies were calibrated by the C1*s* peak (284.8 eV).

### Time-of-flight secondary-ion mass spectrometry (TOF-SIMS) experiments

After the Si/CoO_x_/KBi_(aq)_/MnO_x_ devices were photo charged and dark discharged under zero bias for 100 s in KBi solution with H_2_^18^O and D_2_O solvents, the samples were analyzed by TOF-SIMS (Münster, Germany) with a detection mode of negative ions. The Bi^+^ ions with 30 keV was used for TOF analysis in an area of 91 × 91 μm^2^, a beam of 1 keV Cs^+^ ions for sputter etching in an area of 250 × 250 μm^2^.

### Electrochemical and photoelectrochemical measurements

For half-cell measurement, the Si/CoO_x_, a saturated calomel electrode and a graphite rod were used as a working electrode, a reference electrode, and a counter electrode, respectively. The electrolyte was KBi solution (0.2 M KOH and 0.4 M H_3_BO_3_) with pH = 9. The light source was 1 Sun of simulated solar illumination by a Xe lamp with AM 1.5 G filter (100 mW cm^−2^). Cyclic voltammetry (CV) curves, galvanostatic charge-discharge (GCD) curves and electrochemical impedance spectroscopy (EIS) were recorded using an electrochemical workstation (CHI 760E, Shanghai Chenhua). The areal charge quantity (*Q*) and energy density (*E*) of a solar rechargeable device were calculated following Eqs. (1) and (2), respectively, where *I* is the charge/discharge current under zero bias, t is the charge/discharge time, and Δ*V* is the voltage difference of dark discharge^23^.

1 $$Q=\int I\,{dt}$$

2 $$E=Q\Delta V$$

## Supplementary information

Supplementary Information

Description of Additional Supplementary Files

Supplementary Movie 1

## Data Availability

All data are available in the main text or the Supplementary Information files. Additional data related to the findings of this study are available from the corresponding author upon reasonable request.
