# Supplementary material for: Photovoltage memory effect in a portable Faradaic junction solar rechargeable device
Source: Nat Commun. 2022 May 10;13:2544. doi: 10.1038/s41467-022-30346-z (PMC9090830; doi:10.1038/s41467-022-30346-z)
Supplement: Supplementary file 1 — Supplementary Information [file 41467_2022_30346_MOESM1_ESM.pdf]

Supplementary Information

Photovoltage Memory Effect in a Portable Faradaic Junction Solar Rechargeable Device

Pin Wang[1], Mengfan Xue[1], Dongjian Jiang[2], Yanliang Yang[2], Junzhe Zhang[2], Hongzheng Dong[2], Gengzhi Sun[3], Yingfang Yao[2], Wenjun Luo[2]*, Zhigang Zou[1,2]

[1]Eco-materials and Renewable Energy Research Center (ERERC), Jiangsu Key Laboratory for Nano Technology, National Laboratory of Solid State Microstructures and Department of Physics, Nanjing University, Nanjing 210093, China

[2]College of Engineering and Applied Sciences, Nanjing University, Nanjing 210093, China

[3]Key Laboratory of Flexible Electronics (KLOFE) & Institute of Advanced Materials (IAM), Nanjing Tech University, Nanjing 211816, China

*Email: wjluo@nju.edu.cn;

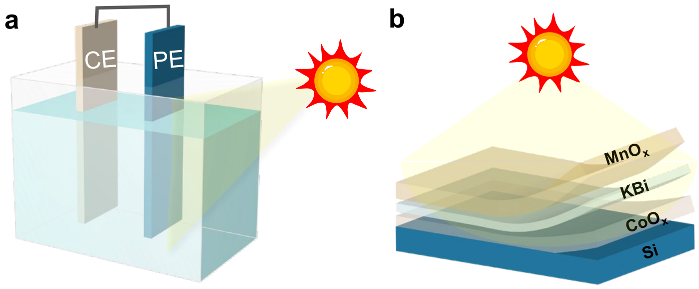

Supplementary Fig. 1 Schematic illustrations of two kinds of solar rechargeable devices. **a**, A two-electrode solar rechargeable device with an opaque carbon counter electrode. PE: photoelectrode; CE: counter electrode. **b**, Configuration of a portable $Si/CoO_x/KBi_{(aq)}/MnO_x$ device.

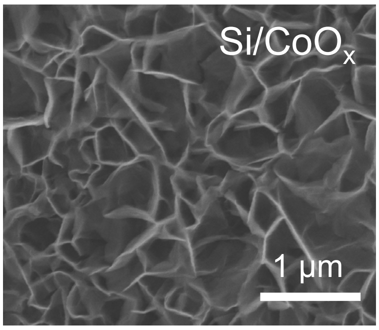

Supplementary Fig. 2 Surface SEM image of a Si/CoO_x photoelectrode. The Si/CoO_x photoelectrode was obtained by pretreatment at -0.3 V_{SCE} to 0.6 V_{SCE} for 25 cycles in KBi solution (0.2 M KOH and 0.4 M H_3BO_3) with pH=9 under illumination.

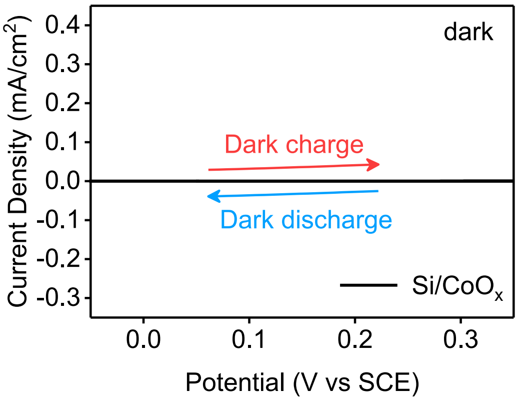

Supplementary Fig. 3 The electrochemical property of a Si/CoO_x sample. CV curve of Si/CoO_x at the scan rate of 10 mV/s in the dark. Electrolyte: KBi aqueous solution (0.2 M KOH and 0.4 M H_3BO_3) with pH=9.

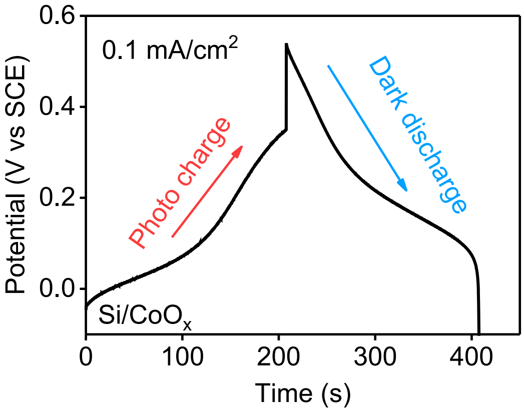

Supplementary Fig. 4 GCD curve of a Si/CoO_x photoelectrode during photo charge and dark discharge. The jump of potential at 208 s comes from the disappearance of

photovoltage when the light is off. Light source: 1 Sun of simulated solar illumination by a Xe lamp with AM 1.5G filter (100 mW/cm^2), electrolyte: KBi aqueous solution (0.2 M KOH and 0.4 M H_3BO_3) with pH=9.

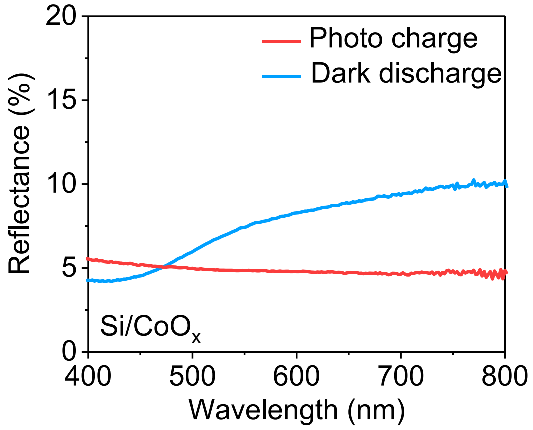

Supplementary Fig. 5 UV-Vis spectra of Si/CoO_x photoelectrodes after photo charge and dark discharge. The charging-discharging processes were performed in a three-electrode setup by chronopotentiometry method. Light source: 1 Sun of simulated solar illumination by a Xe lamp with AM 1.5G filter (100 mW/cm^2), electrolyte: KBi aqueous solution (0.2 M KOH and 0.4 M H_3BO_3) with pH=9.

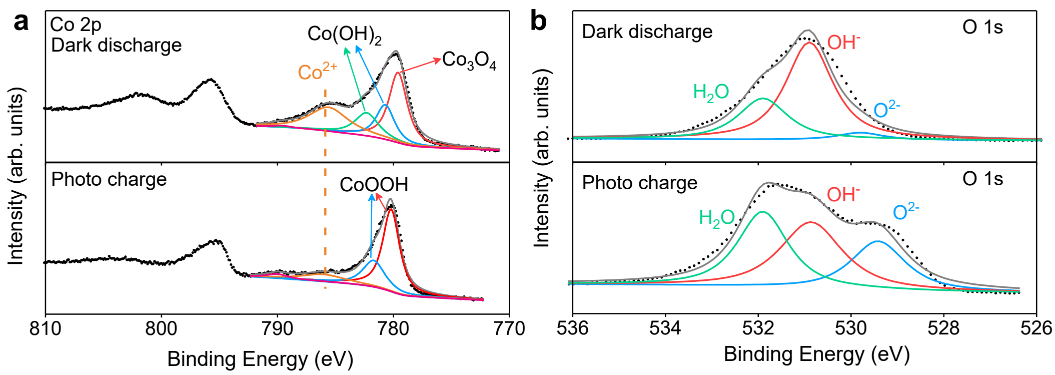

Supplementary Fig. 6 XPS spectra for Si/CoO_x photoelectrodes at the stages of dark discharge and photo charge. **a**, XPS of Co 2*p* core level. After dark discharge, the binding energies of Co $2p_{3/2}$ are observed at 779.6 eV assigned to Co_3O_4, 780.7 eV and 782.3 eV to $Co(OH)_2$. A satellite peak corresponding to Co^{2+} is also observed at 786

eV[1]. After photo charge, the binding energies at 780.2 eV and 781.7 eV correspond to CoOOH[1,2]. **b,** XPS of O 1*s* core level, three peaks at 529.4 eV, 530.9 eV and 531.9 eV are assigned to lattice O^{2-}, lattice OH^- and adsorbed H_2O molecules, respectively[3].

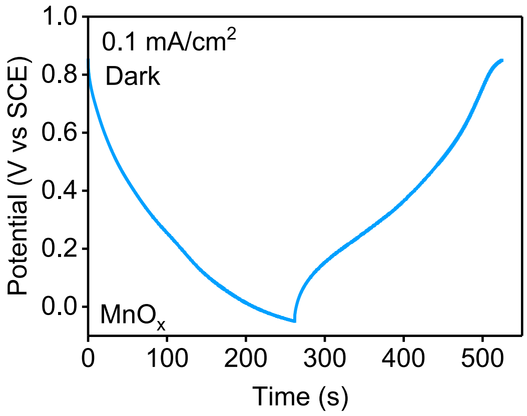

Supplementary Fig. 7 GCD curve of a MnO_x counter electrode. Electrolyte: KBi aqueous solution (0.2 M KOH and 0.4 M H_3BO_3) with pH=9.

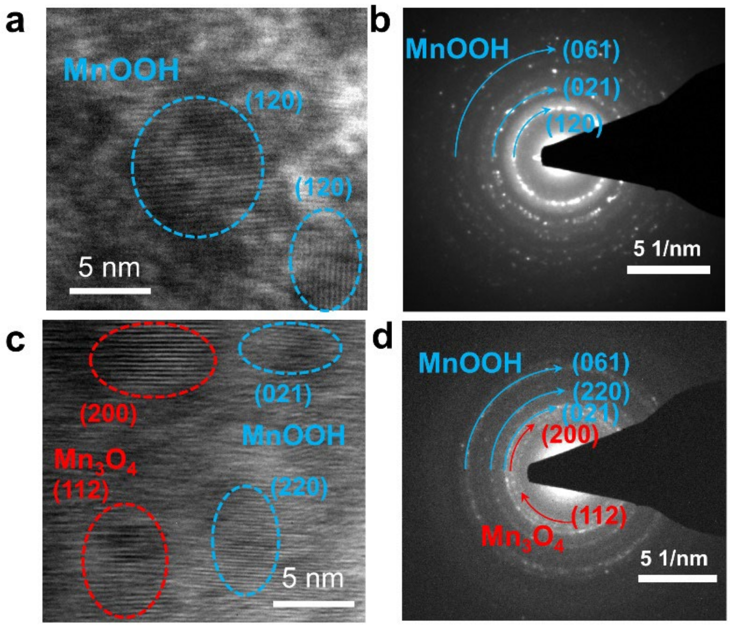

Supplementary Fig. 8 The characterization of MnO_x counter electrodes during dark discharge and dark charge. **a, b, c, d,** TEM images and the corresponding FFT patterns of MnO_x counter electrodes after dark discharge (**a, b**) and after dark charge (**c, d**).

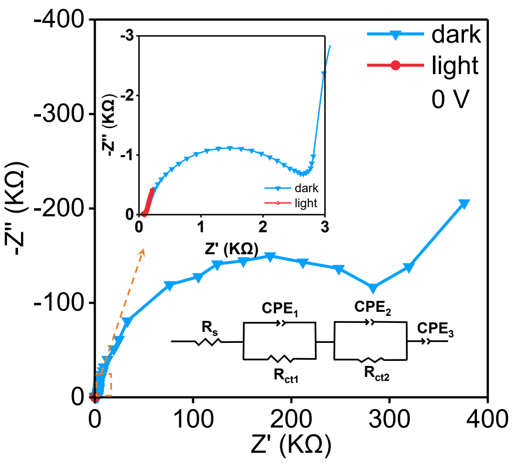

Supplementary Fig. 9 Nyquist plots and an equivalent circuit of the $Si/CoO_x/KBi_{(aq)}/MnO_x$ device without bias under illumination and in the dark. Light source: 1 Sun of simulated solar illumination by a Xe lamp with AM 1.5G filter (100 mW/cm^2), electrolyte: KBi aqueous solution (0.2 M KOH and 0.4 M H_3BO_3) with pH=9, frequency range: 0.1-100000 Hz. R_s represents series resistance. R_{ct1} and CPE_1 represent the charge-transfer resistance and the capacitance of semiconductor/Faradaic material interface. R_{ct2} and CPE_2 represent the charge-transfer resistance and double layer capacitance of Faradaic material/electrolyte interfaces. CPE_3 is the pseudocapacitance corresponding to the Faradaic material/electrolyte interfaces.[4,5]

Supplementary Table 1. The fitted impedance parameters of $Si/CoO_x/KBi_{(aq)}/MnO_x$ under illumination and in the dark from Nyquist plots in Supplementary Fig. 9.

	R_s (Ω)	R_{ct1} (Ω)	R_{ct2} (Ω)	CPE_1 (F)	CPE_2 (F)	CPE_3 (F)
light	52.13	37.54	20.25	$1.66*10^{-6}$	0.007	0.003
dark	56.66	2396	353390	$2.88*10^{-8}$	$7.3*10^{-7}$	$3.5*10^{-6}$

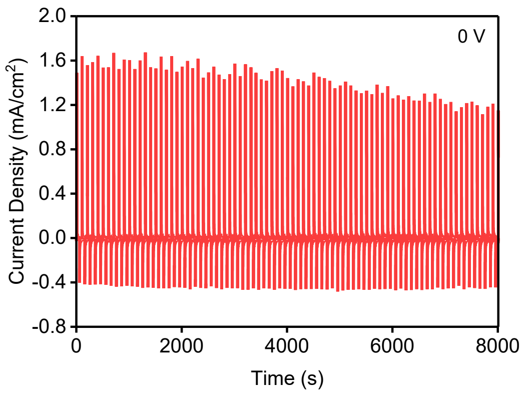

Supplementary Fig. 10 Cyclic stability of Si/CoO_x/$KBi_{(aq)}$/MnO_x during photo charge and dark discharge. Light source: 1 Sun of simulated solar illumination by a Xe lamp with AM 1.5G filter (100 mW/cm^2), electrolyte: KBi aqueous solution (0.2 M KOH and 0.4 M H_3BO_3) with pH=9.

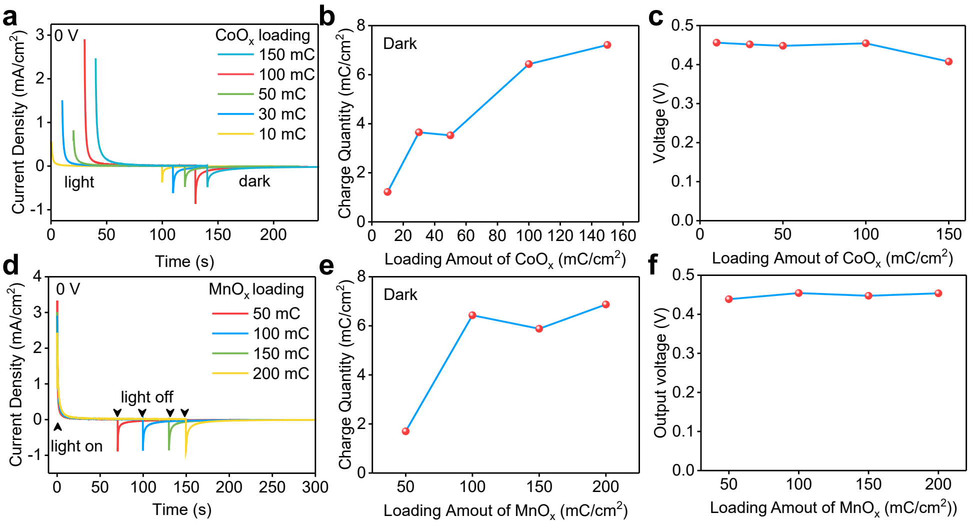

Supplementary Fig. 11 The effect of the loading amount of the Faradaic materials on the photoelectrochemical properties of Si/CoO_x/$KBi_{(aq)}$/MnO_x devices. **a**, **b**, **c**, I-t curves during photo charge and dark discharge under zero bias (**a**), areal charge quantities of dark discharge (**b**), and dark output voltages (**c**) of Si/CoO_x/$KBi_{(aq)}$/MnO_x with different CoO_x loading. **d**, **e**, **f**, I-t curves during photo charge and dark discharge under zero bias (**d**), areal charge quantities of dark discharge (**e**), and dark output voltages (**f**) of Si/CoO_x/$KBi_{(aq)}$/MnO_x with different MnO_x loading. Light source: 1 Sun of simulated solar illumination by a Xe lamp with AM 1.5G filter (100 mW/cm^2), electrolyte: KBi aqueous solution (0.2 M KOH and 0.4 M H_3BO_3) with pH=9.

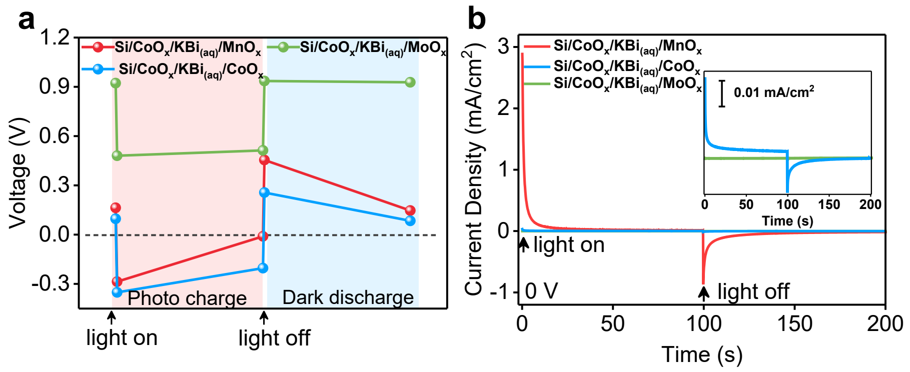

Supplementary Fig. 12 The effects of the photovoltage memory effect on the performance. **a**, Open circuit voltages of the three devices, and the photo charge and dark discharge were carried out in the three devices by i-t curves without bias. **b**, I-t curves of Si/CoO_x/$KBi_{(aq)}$/MnO_x, Si/CoO_x/$KBi_{(aq)}$/CoO_x and Si/CoO_x/$KBi_{(aq)}$/MoO_x full cells during photo charge and dark discharge under zero bias. The loading amount of CoO_x on Si, MnO_x, CoO_x, and MoO_x counter electrodes is 100 mC/cm^2. Light source: 1 Sun of simulated solar illumination by a Xe lamp with AM 1.5G filter (100 mW/cm^2), electrolyte: KBi aqueous solution (0.2 M KOH and 0.4 M H_3BO_3) with pH=9.

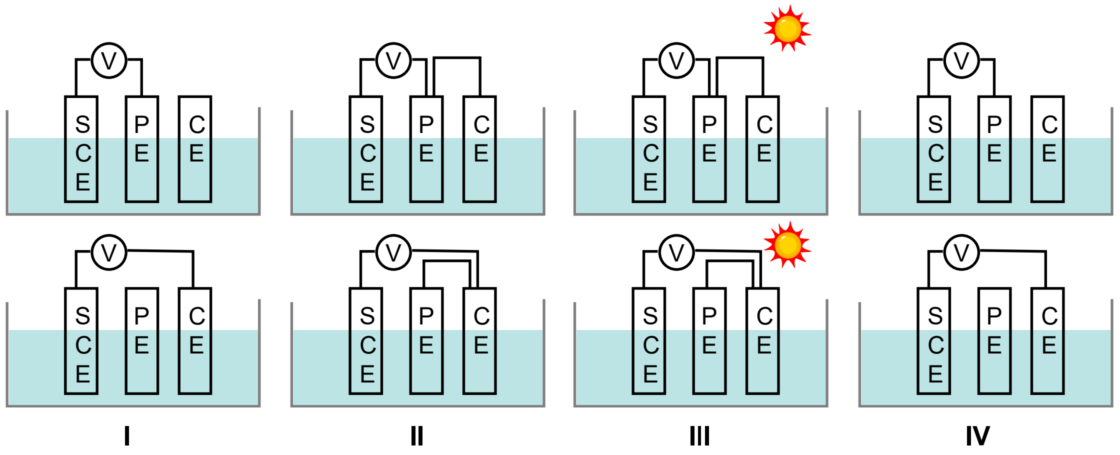

Supplementary Fig. 13 Electrical connection modes for open circuit potential (OCP) measurement at different working stages, I: disconnect-dark stage; II: connect-dark stage; III: connect-light stage; IV: disconnect-dark stage. SCE, PE and CE are a saturated calomel electrode, a photoelectrode and a counter electrode, respectively.

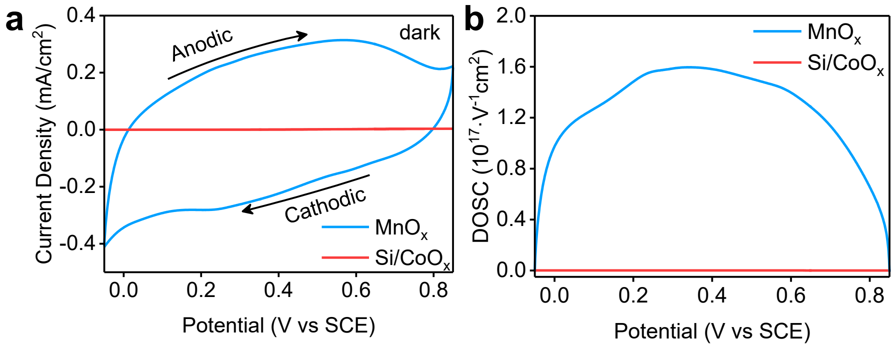

Supplementary Fig. 14 The measurement and calculation method of densities of storable charge (DOSC) of MnO_x and Si/CoO_x in the dark. **a**, CV curves with the scan rate of 10 mV/s. **b**, DOSC is obtained from **a**. The DOSC (V^{-1} cm^{-2}) is calculated by $\frac{dN}{dV} = \frac{\frac{dQ}{e}}{dV} = \frac{dQ}{e \cdot dV} = \frac{\frac{I_a - I_c}{2} \cdot dt}{e \cdot dV} = \frac{\frac{I_a - I_c}{2}}{e \cdot dV/dt} = \frac{I_a - I_c}{2e \cdot v}$, where N is the storable charge number, V is the potential (V), S is the electrode area (cm^2), Q is the areal charge quantity (mC cm^{-2}), e is the electron charge, I_a is the anodic current density (mA cm^{-2}), I_c is the cathodic current density (mA cm^{-2}), and v is the scan rate (V s^{-1}). Electrolyte: KBi aqueous solution (0.2 M KOH and 0.4 M H_3BO_3) with pH=9.

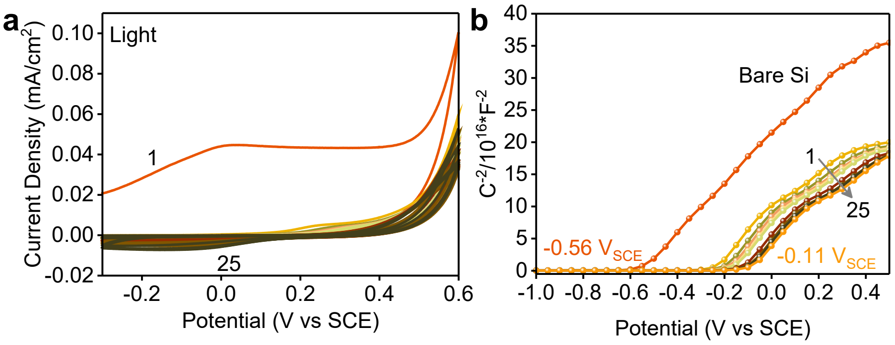

Supplementary Fig. 15 The flat-band potential shifts with different CV cycles on a bare Si. **a**, The anodic cycling activation process of a bare Si under illumination. **b**, Mott-Schottky plots of a bare Si without and with anodic cycling activation process in the dark. Light source: 1 Sun of simulated solar illumination by a Xe lamp with AM 1.5G filter (100 mW/cm^2), electrolyte: KBi aqueous solution (0.2 M KOH and 0.4 M H_3BO_3) with pH=9.

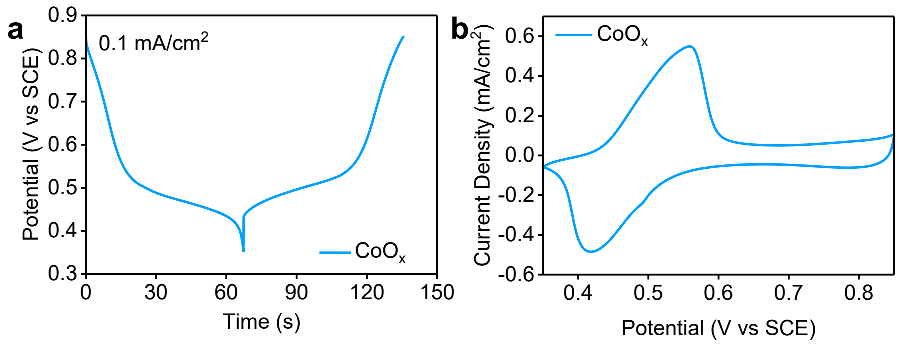

Supplementary Fig. 16 The electrochemical properties of a CoO_x counter electrode. **a**, GCD curve of a CoO_x counter electrode at the current density of 0.1 mA/cm^2 in the dark. **b**, CV curve of a CoO_x counter electrode at the scan rate of 10 mV/s in the dark. Electrolyte: KBi aqueous solution (0.2 M KOH and 0.4 M H_3BO_3) with pH=9.

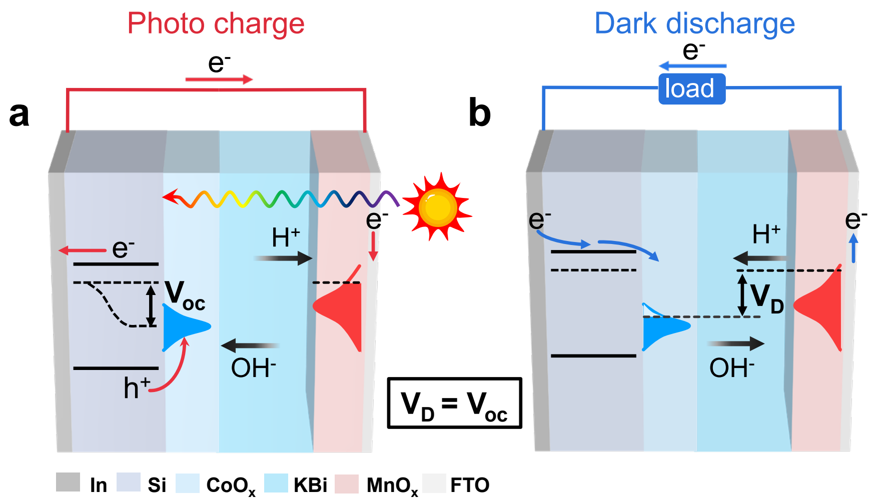

Supplementary Fig. 17 A mechanism schematic of a Faradaic junction solar rechargeable device with the photovoltage memory effect. **a**, Photo charge process. **b**, Dark discharge process.

During photo charge, the Si/CoO_x photoelectrode is connected with the MnO_x counter electrode in short circuit. The photo-generated holes in the Si semiconductor transfer and are stored in the CoO_x layer, while the photo-generated electrons transfer and are stored in the MnO_x counter electrode. At the same time, OH^- and H^+ transfer into the CoO_x and MnO_x to balance the charge. The photo charge process can adjust the potentials of CoO_x and MnO_x. When the hole and electron quasi-Fermi levels in Si are

the same with the potentials of CoO_x and MnO_x, the photo charge process will end (Supplementary Fig. 17a). In this case, the electron and hole quasi-Fermi levels in Si under illumination are exactly recorded by MnO_x and CoO_x, respectively. When the light is off, the potentials of MnO_x and CoO_x after photo charge can be kept since both of them indicate high Faradaic capacitance. Therefore, the dark output voltage (V_D) is equal to the photovoltage (V_{oc}), which is the photovoltage memory effect.

During dark discharge, the stored electrons in MnO_x transfer back to CoO_x in the photoelectrode through a load, which can make the potentials of MnO_x and CoO_x after photo charge return to the initial values before photo charge (Supplementary Fig. 17b). Therefore, a photo charge/dark discharge cycle completes in the two-electrode device.

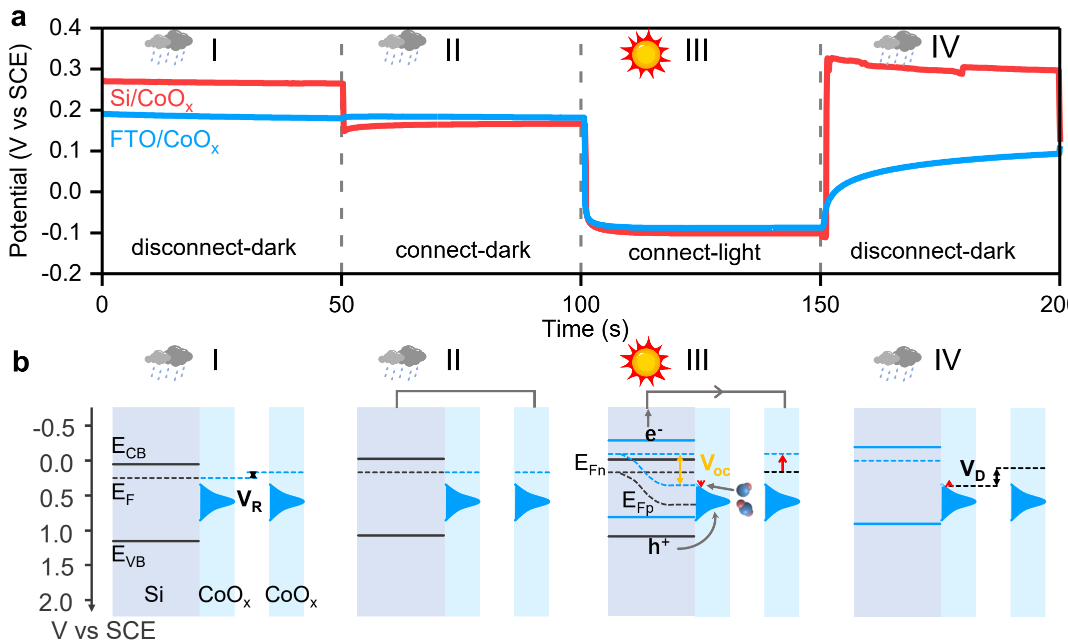

Supplementary Fig. 18 The charge transfer mechanism in a $Si/CoO_x/KBi_{(aq)}/CoO_x$ device. **a**, OCPs of a Si/CoO_x photoelectrode and a CoO_x counter electrode under illumination and in the dark. The OCPs were measured under disconnected and connected modes. Light source: 1 Sun of simulated solar illumination by a Xe lamp with AM 1.5G filter (100 mW/cm^2), electrolyte: KBi aqueous solution (0.2 M KOH and 0.4 M H_3BO_3) with pH=9. **b**, Energy band diagrams of $Si/CoO_x/KBi_{(aq)}/CoO_x$ at different working stages. E_{VB} and E_{CB} are the valence band and conduction band of a

semiconductor, respectively; E_{Fn} and E_{Fp} represent the quasi-Fermi levels of electrons and holes, respectively; V_R is a residue voltage between a photoelectrode and a counter electrode; V_{oc} represents a photovoltage and V_D represents a dark output voltage.

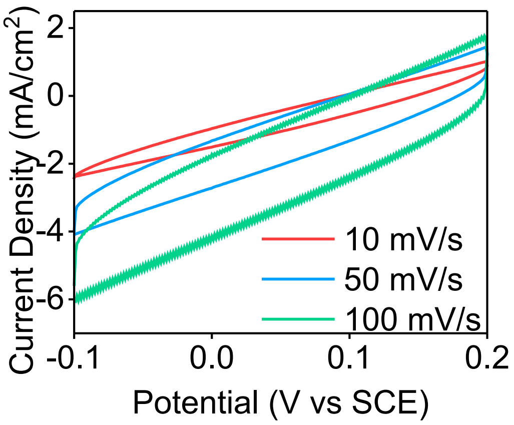

Supplementary Fig. 19 CV curves of a CoO_x counter electrode at the scan rates of 10, 50 and 100 mV/s in the dark. Electrolyte: KBi aqueous solution (0.2 M KOH and 0.4 M H_3BO_3) with pH=9.

The OCP method and band diagrams are used to understand on different photoelectrochemial properties in the Si/CoO_x/$KBi_{(aq)}$/CoO_x device (Supplementary Fig. 18). Similar to Si/CoO_x/$KBi_{(aq)}$/MnO_x, after the photoelectrode is connected with the counter electrode by a Cu wire in the dark, the OCP of the Si/CoO_x photoelectrode is adjusted to the OCP of the CoO_x counter electrode. When the light is on, the OCPs of the photoelectrode and the counter electrode decrease quickly from 0.17 V to -0.1 V. After the two electrodes are disconnected and the light is off, the OCP of Si/CoO_x jumps to 0.33 V, while the CoO_x counter electrode indicates much faster potential recovery process than the MnO_x counter electrode (Fig. 3a, Stage IV). The equilibrium potential of MnO_x is in the Faradaic potential window, while the equilibrium potential of CoO_x is negative than the Faradaic window of CoO_x and in the electric double layer (EDL) window of CoO_x (Supplementary Fig. 19). Therefore, the EDL of CoO_x counter electrode is charged by photo-generated electrons from Si, while the Faradaic layer of MnO_x counter electrode is photo charged. The EDL has much shorter storage time and

less charge quantity than the Faradaic layer[6,7], which leads to faster recovery of the potential of the CoO_x counter electrode than the MnO_x counter electrode. Therefore, the severe dark output voltage loss and less stored charges in Fig. 2d-f are observed in $Si/CoO_x/KBi_{(aq)}/CoO_x$ than $Si/CoO_x/KBi_{(aq)}/MnO_x$.

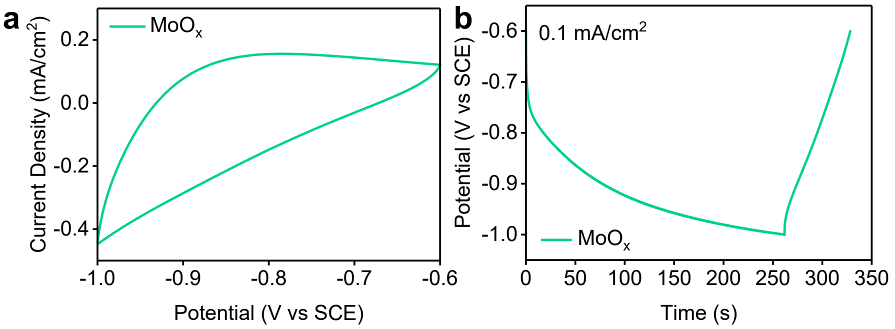

Supplementary Fig. 20 The electrochemical properties of a MoO_x counter electrode. **a**, CV curve at the scan rate of 10 mV/s. **b**, GCD curve at the current density of 0.1 mA/cm^2. Electrolyte: KBi aqueous solution (0.2 M KOH and 0.4 M H_3BO_3) with pH=9.

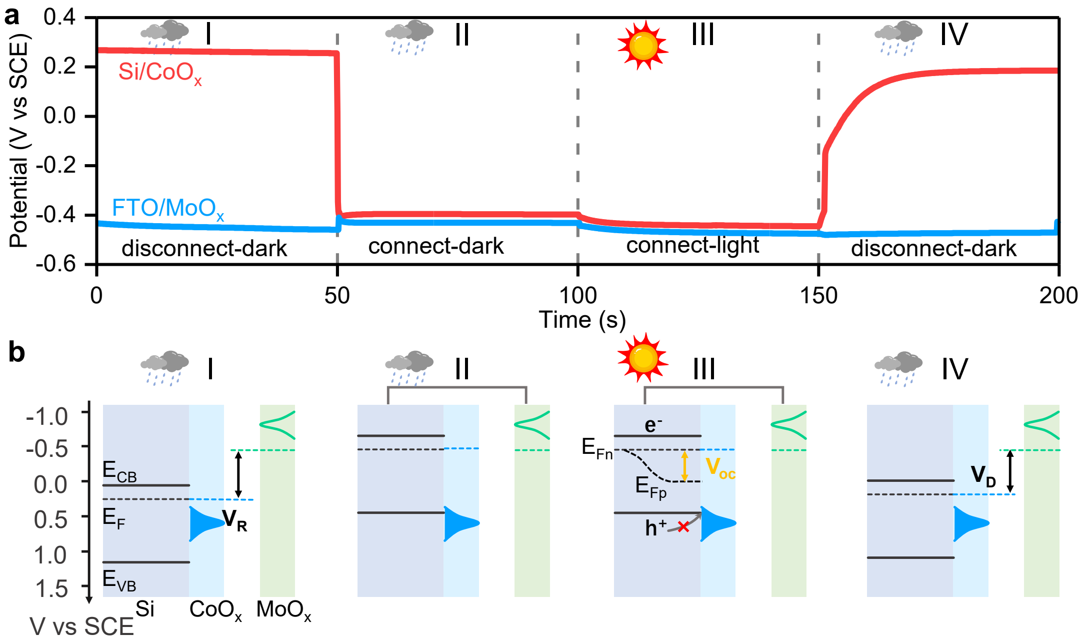

Supplementary Fig. 21 The charge transfer mechanism in a $Si/CoO_x/KBi_{(aq)}/MoO_x$ device. **a**, OCPs of a Si/CoO_x photoelectrode and a MoO_x counter electrode under illumination and in the dark. The OCPs were measured under disconnected and

connected modes. Light source: 1 Sun of simulated solar illumination by a Xe lamp with AM 1.5G filter (100 mW/cm^2), electrolyte: KBi aqueous solution (0.2 M KOH and 0.4 M H_3BO_3) with pH=9. **b**, Energy band diagrams of Si/CoO_x/$KBi_{(aq)}$/MoO_x at different working stages. E_{VB} and E_{CB} are the valence band and conduction band of a semiconductor, respectively; E_{Fn} and E_{Fp} represent the quasi-Fermi levels of electrons and holes, respectively; V_R is a residue voltage between a photoelectrode and a counter electrode; V_{oc} represents a photovoltage and V_D represents a dark output voltage.

For the Si/CoO_x/$KBi_{(aq)}$/MoO_x device, the Faradaic window and equilibrium potential of the MoO_x counter electrode were measured and the results are shown in Supplementary Figs. 20 and 21. The MoO_x indicates much negative equilibrium potential than the MnO_x and CoO_x counter electrode, which leads to higher residue voltage (V_R) of 0.94 V in Si/CoO_x/$KBi_{(aq)}$/MoO_x than the Si/CoO_x/$KBi_{(aq)}$/MnO_x and Si/CoO_x/$KBi_{(aq)}$/CoO_x (Supplementary Fig. 21). If the residue voltage of the device is higher than the photovolatge of the Faradaic junction, the photogenerated holes will not oxidize CoO_x (Supplementary Fig. 21), which causes that the device cannot be photo-charged under zero bias. Therefore, the charge quantity of photo charge and dark discharge is negligible (Fig. 2e) and the dark output voltage keeps the residue voltage before illumination.

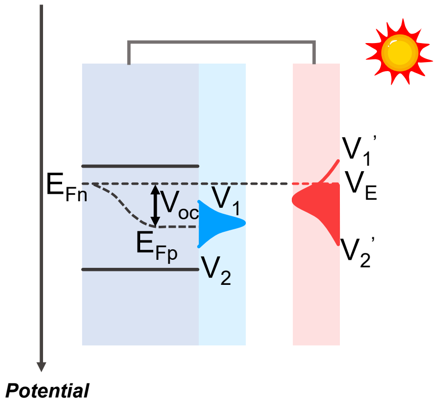

Supplementary Fig. 22 Schematic of working prerequisite for the photovoltage memory effect in a solar rechargeable device. V_1 and V_2 are a lower and upper limit of

Faradaic potential window of a faradaic material in a photoelectrode, respectively; V_1' and V_2' are a lower and upper limit of Faradaic potential window of a counter electrode, respectively; V_E is an equilibrium potential of a counter electrode. V_{oc} is a photovoltage in a photoelectrode. To realize a photo-oxidization of a Faradaic material on a semiconductor, the hole quasi-Fermi level in a semiconductor should be positive than V_1 and negative than V_2, that is, $V_1 < V_E + V_{oc} < V_2$. On the other hand, if photo-generated electrons from a semiconductor can reduce the Faradaic layer, not the electric double layer of a counter electrode, V_E should be positive than V_1' and negative than V_2' ($V_1' < V_E < V_2'$).

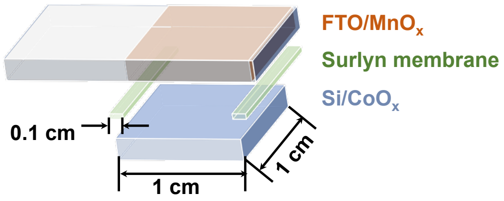

Supplementary Fig. 23 Diagram of a portable Si/CoO_x/$KBi_{(aq)}$/MnO_x device. The length and the width of the surlyn membrane are about 1 cm and 0.1 cm, respectively. The sizes of the Si/CoO_x photoelectrode and the FTO/MnO_x counter electrode are both 1*1 cm^2. Therefore, the effective area of the device is about 0.8 cm^2.

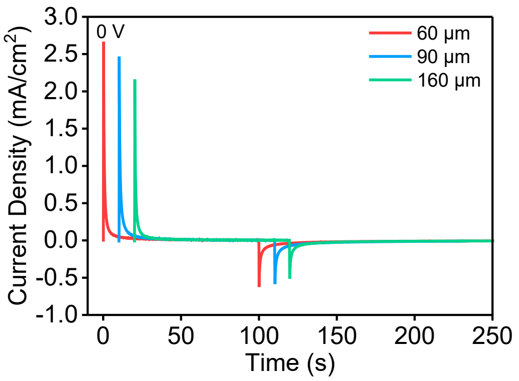

Supplementary Fig. 24 The effects of the membrane thickness on the performance of the Si/CoO_x/$KBi_{(aq)}$/MnO_x Faradaic junction device. I-t curves during photo charge and dark discharge of Si/CoO_x/$KBi_{(aq)}$/MnO_x with the surlyn membrane thickness of 60, 90 and 160 μm.

Supplementary Table 2. The output performance comparison of two-electrode solar rechargeable devices without bias in this study and previous literatures. Q_{output} represents areal charge quantity during dark discharge. V_{output} represents dark output voltage. E_{output} represents dark volumetric energy density. CE represents coulomb efficiency and stability is characterized by the cycles of photo charge and dark discharge.

Two-electrode Devices	Q_{output} (mC/cm^2)	V_{output} (V)	E_{output} (mJ/cm^3)	CE	Stability (cycle)	Ref.
TiO_2-dye-LiI/AC/ Polymer electrolyte/AC/Pt	negligible	0.45	negligible	N/A	N/A	8
TiO_2/NiO/$Na_2SO_{4(aq)}$/Pt/Si	negligible	0.45	negligible	N/A	N/A	9
Si/Porous Si/Polymer electrolyte/Porous Si	negligible	0.55	negligible	N/A	N/A	10
Si/WO_3/$H_2SO_{4(aq)}$/C	8.60	0.50	0.06 (2*4*6 cm^3)	88%	5	11
Si/CoO_x/$Kbi_{(aq)}$/MnO_x	6.53	0.45	1.89 (1*2*0.5 cm^3)	100%	80	This work

Supplementary References

1. Yang, J. *et al.* Efficient and sustained photoelectrochemical water oxidation by cobalt oxide/silicon photoanodes with nanotextured interfaces. *J. Am. Chem. Soc.* **136**, 6191-6194 (2014).
2. Tian, L. *et al.* Synthesis and characterization of α-cobalt hydroxide nanobelts. *J. Phys. Chem. C* **114**, 111-119 (2010).
3. Yin, Z. *et al.* Mildly regulated intrinsic faradaic layer at the oxide/water interface for improved photoelectrochemical performance. *Chem. Sci.* **11**, 6297-6304 (2020).
4. Chen, C., Lu, Y., Fan, R. & Shen, M. Integration of Oxygen-Vacancy-Rich NiFe-Layered Double Hydroxide onto Silicon as Photoanode for Enhanced Photoelectrochemical Water Oxidation. *ChemSusChem* **13**, 3893-3900 (2020).
5. Gao, Z. *et al.* Graphene nanosheet/Ni^{2+}/Al^{3+} layered double-hydroxide composite as a novel electrode for a supercapacitor. *Chem. Mater.* **23**, 3509-3516

(2011).

6. Shao, Y. *et al.* Design and Mechanisms of Asymmetric Supercapacitors. *Chem. Rev.* **118**, 9233-9280 (2018).
7. Rivnay, J. *et al.* Organic electrochemical transistors. *Nat. Rev. Mater.* **3**, (2018).
8. Miyasaka, T. & Murakami, T. N. The photocapacitor: An efficient self-charging capacitor for direct storage of solar energy. Appl. Phys. Lett. 85, 3932-3934 (2004).
9. Wang, Y. *et al.* Fully solar-powered photoelectrochemical conversion for simultaneous energy storage and chemical sensing. *Nano Lett.* **14**, 3668-3673 (2014).
10. A. S. Westover, *et al.*, Direct integration of a supercapacitor into the backside of a silicon photovoltaic device. *Appl. Phys. Lett.* **104**, 1-4 (2014).
11. Wang, P. *et al.* A Capacitor-type Faradaic Junction for Direct Solar Energy Conversion and Storage. *Angew. Chem. Int. Ed.* **60**, 1390-1395 (2021).
