# Supplementary material for: Photovoltage memory effect in a portable Faradaic junction solar rechargeable device
Source: Nat Commun. 2022 May 10;13:2544. doi: 10.1038/s41467-022-30346-z (PMC9090830; doi:10.1038/s41467-022-30346-z)
Supplement: Supplementary file 2 — Description of Additional Supplementary Files [file 41467_2022_30346_MOESM2_ESM.pdf]

Description of Additional Supplementary Files

File name: Supplementary Movie 1

Description: Demonstration of portable solar rechargeable devices for lighting a LED bulb after photo charge. Five Si/CoO_x/$KBi_{(aq)}$/MnO_x devices are connected in series.
